# Using Non-linear Homogenization to Improve the Performance of Macroscopic Damage Models of Trabecular Bone

**DOI:** 10.3389/fphys.2018.00545

**Published:** 2018-05-17

**Authors:** Francesc Levrero-Florencio, Pankaj Pankaj

**Affiliations:** ^1^Computational Cardiovascular Science, Department of Computer Science, University of Oxford, Oxford, United Kingdom; ^2^Institute for Bioengineering, School of Engineering, The University of Edinburgh, Edinburgh, United Kingdom

**Keywords:** trabecular bone, multiscale modeling, parameter estimation, continuum damage, finite element method, homogenization, biomechanics, high performance computing

## Abstract

Realistic macro-level finite element simulations of the mechanical behavior of trabecular bone, a cellular anisotropic material, require a suitable constitutive model; a model that incorporates the mechanical response of bone for complex loading scenarios and includes post-elastic phenomena, such as plasticity (permanent deformations) and damage (permanent stiffness reduction), which bone is likely to experience. Some such models have been developed by conducting homogenization-based multiscale finite element simulations on bone micro-structure. While homogenization has been fairly successful in the elastic regime and, to some extent, in modeling the macroscopic plastic response, it has remained a challenge with respect to modeling damage. This study uses a homogenization scheme to upscale the damage behavior from the tissue level (microscale) to the organ level (macroscale) and assesses the suitability of different damage constitutive laws. Ten cubic specimens were each subjected to 21 strain-controlled load cases for a small range of macroscopic post-elastic strains. Isotropic and anisotropic criteria were considered, density and fabric relationships were used in the formulation of the damage law, and a combined isotropic/anisotropic law with tension/compression asymmetry was formulated, based on the homogenized results, as a possible alternative to the currently used single scalar damage criterion. This computational study enhances the current knowledge on the macroscopic damage behavior of trabecular bone. By developing relationships of damage progression with bone's micro-architectural indices (density and fabric) the study also provides an aid for the creation of more precise macroscale continuum models, which are likely to improve clinical predictions.

## 1. Introduction

The growth of older population around the world in the last few decades has caused an increase in problems which can be associated to deteriorated mechanical properties of bone; osteoporosis is the clearest example of one such condition.

Computer models have been extensively employed to evaluate the mechanical response of bone and bone-implant systems under a range of loading scenarios (Pankaj, 2013). Previous studies have assumed bone to be homogeneous (Completo et al., 2009; Conlisk et al., 2015), i.e., its properties do not vary from point to point in space or heterogeneous (Helgason et al., 2008; Schileo et al., 2008; Tassani et al., 2011), i.e., its properties vary with location (these are typically assigned on the basis of grey-scale values observed in micro-computed tomography scans). However, in the large majority of studies, bone is assumed to be linear elastic and isotropic, i.e. its properties at a certain point in space are the same in all directions. It is well- recognized that the cellular microstructure of trabecular bone renders it anisotropic (Turner et al., 1990; Odgaard et al., 1997), i.e., properties at a point in space vary in different directions. Finite element (FE) analysis of the bone microstructure, in which the solid and pore phases are explicitly modeled, has been used to evaluate the homogenized anisotropic linear elastic properties of bone in the past two decades. Morphology-elasticity relationships that use bone density and fabric have also been established, with fabric typically measured through the mean intercept length (MIL) fabric tensor (Harrigan and Mann, 1984). These relationships establish links between density, fabric, and the components of the stiffness tensor (Zysset, 2003). More recently, some studies have attempted the evaluation of homogenized yield behaviour (Cowin, 1986; Wolfram et al., 2012; Levrero-Florencio et al., 2016).

Homogenized FE models of the whole bone can include microstructural information at the continuum (macroscopic) level and can thus improve the assessment of the behavior of bone and bone-implant systems in clinical scenarios. Homogenization relies on averaging the strains and stresses over a representative volume element (RVE) of the considered material; it is the most widely used multiscale approach to study the macroscopic behavior of trabecular bone. Homogenization of an RVE in the post-elastic regime requires examining its response to a wide range of loading scenarios (Bayraktar et al., 2004; Levrero-Florencio et al., 2016, 2017a). It is important to note that, in experiments, it is not possible to test multiple load cases after a certain load threshold has been surpassed because permanent deformations and/or damage caused during the first loading case will affect the behavior in subsequent loading cases. Therefore, computational means provide an attractive alternative. Nonetheless, the need for fine resolution to recreate a biofidelic geometry of the bone microstructure leads to micro-FE (μFE) systems of several tens of millions of degrees of freedom. The need to undertake multiple load cases each in non-linear regime requires the usage of high performance computing (HPC) platforms and software which can take advantage of them.

Although the damage behavior of bone has been considered in a few studies (Keaveny et al., 1994a; Garcia et al., 2009; Shi et al., 2010; Schwiedrzik and Zysset, 2013; Lambers et al., 2014), there are apparent limitations to most of the employed mathematical formulations. For example, most macroscopic damage models of trabecular bone employ an isotropic damage evolution, i.e., a “basic,” or single scalar isotropic formulation, as mentioned in Carol et al. (2002), and do not take into account that the development of damage may be related to the load case being considered (Levrero-Florencio et al., 2017a). The authors have previously conducted a series of uniaxial simulations which show that damage develops differently in tension−compression, and in normal−shear (Levrero-Florencio et al., 2017a).

This study has a number of aims. Firstly, it extends the study performed in Levrero-Florencio et al. (2017a) by adding 12 biaxial macroscopic cases in the normal strain space. The second aim is to examine the suitability of certain damage mechanisms by fitting different damage laws to the damaged macroscopic stiffness tensors. The study then investigates the possible relationships between the macroscopic damage behavior of trabecular bone and its density and fabric description, by including these micro-architectural indices as additional data in the fitting procedure. The data for these formulations is obtained computationally through homogenization-based multiscale simulations run on a HPC platform with an *in-house* developed parallel implicit FE code.

## 2. Notation

The mathematical operators defined in this section largely follow the notation used in Wu and Li (2008), Schwiedrzik et al. (2013), and Levrero-Florencio et al. (2016). Compact tensor notation is used throughout this study, with indicial notation within brackets being used in this section to clarify certain tensorial operations, or in specific sections where further clarification might be required.

As a general rule, scalars are denoted with Greek or Latin italic characters (e.g., λ or *a*, respectively); vectors, or first-order tensors, are denoted by Latin bold lower-case characters (e.g., **a**); second-order tensors are denoted with Greek or Latin bold upper-case characters (e.g., σ or **A**, respectively); and fourth-order tensors are denoted by Latin double-barred upper-case characters (e.g., 𝔸).

Tensorial operations are denoted as follows. Single contraction of tensorial entities may appear as **a**·**b** (*a*_*i*_*b*_*i*_), **a**·**B** (*a*_*i*_*B*_*ij*_), **Ab** (*A*_*ij*_*b*_*j*_), or **AB** (*A*_*ik*_*B*_*kj*_), note that the scalar product symbol (·) only appears when the first entity to be contracted is a first-order tensor; double contraction of tensorial entities may appear as **A**:**B** (*A*_*ij*_*B*_*ij*_), 𝔸:**B** (*A*_*ijkl*_*B*_*kl*_), **A**:𝔹 (*A*_*ij*_*B*_*ijkl*_), or 𝔸 : 𝔹 (*A*_*ijmn*_*B*_*mnkl*_). Different tensor products have been defined, which include **a** ⊗ **b** (*a*_*i*_*b*_*j*_), **A** ⊗ **B** (*A*_*ij*_*B*_*kl*_), **A**⊗**B** (*A*_*ik*_*B*_*jl*_), **A**$\bar{\underline{⊗}}$**B** (*A*_*il*_*B*_*jk*_), or $A\underline{\bar{⊗}}B=\frac{1}{2}(A\underline{⊗}B+A\bar{⊗}B)$ ($\frac{1}{2}\left[A_{ik}B_{jl}+A_{il}B_{jk}\right]$).

Curly brackets {·} are used to represent vector projections of second-order tensors, such as

(1) $$\{A\}={\left\{\begin{array}{cccccc} A_{11} & A_{22} & A_{33} & A_{12} & A_{13} & A_{23} \end{array}\right\}}^{\text{T}}.$$

Square brackets [·] are used, in conjunction with parentheses (·), to indicate priority in the order of mathematical operations; an important exception occurs when square brackets are used to represent the matrix projection of a fourth-order tensor, such as

(2) $$[𝔸]=\left[\begin{array}{cccccc} A_{1111} & A_{1122} & A_{1133} & A_{1112} & A_{1113} & A_{1123}\\ A_{2211} & A_{2222} & A_{2233} & A_{2212} & A_{2213} & A_{2223}\\ A_{3311} & A_{3322} & A_{3333} & A_{3312} & A_{3313} & A_{3323}\\ A_{1211} & A_{1222} & A_{1233} & A_{1212} & A_{1213} & A_{1223}\\ A_{1311} & A_{1322} & A_{1333} & A_{1312} & A_{1313} & A_{1323}\\ A_{2311} & A_{2322} & A_{2333} & A_{2312} & A_{2313} & A_{2323} \end{array}\right].$$

Double vertical bars ||(·)|| are used to represent the Frobenius norm of the matrix (·), such as the Frobenius norm of the following 3 × 3 symmetric matrix,

(3) $$\|[A]\|=\sqrt{A_{11}^{2}+A_{22}^{2}+A_{33}^{2}+2A_{11}^{2}+2A_{13}^{2}+2A_{23}^{2}}.$$

## 3. Materials and methods

### 3.1. Computational methods

This section follows the “Materials and Methods” section in Levrero-Florencio et al. (2017a). The authors used μCT images of trabecular bone samples to create detailed FE models, which ranged from 10 to 30 million elements, representing the solid phase of bone for a cubic trabecular bone samples (which includes both solid phase and pores) of size 5 mm. In the study conducted by Levrero-Florencio et al. (2017a), plasticity and damage were considered for the solid phase post-elastic properties and nine uniaxial strain cases were investigated (load cases 1 to 9 of Table 1) representing: three tensile cases (+ε_11_, +ε_22_, and +ε_33_), three compressive cases (−ε_11_, −ε_22_, and −ε_33_), and three shear cases (ε_12_, ε_13_, and ε_23_). The macroscopic damage behavior was studied by using an appropriate homogenization-based multiscale technique, which is explained later.

**Table 1:** Description of the performed strain-controlled load cases.

**Load case**	**Description**
1	ε_11_ > 0; ε_22_ = ε_33_ = 0
	ε_12_ = ε_13_ = ε_23_ = 0
2	ε_22_ > 0; ε_11_ = ε_33_ = 0
	ε_12_ = ε_13_ = ε_23_ = 0
3	ε_33_ > 0; ε_11_ = ε_22_ = 0
	ε_12_ = ε_13_ = ε_23_ = 0
4	ε_11_ < 0; ε_22_ = ε_33_ = 0
	ε_12_ = ε_13_ = ε_23_ = 0
5	ε_22_ < 0; ε_11_ = ε_33_ = 0
	ε_12_ = ε_13_ = ε_23_ = 0
6	ε_33_ < 0; ε_11_ = ε_22_ = 0
	ε_12_ = ε_13_ = ε_23_ = 0
7	ε_11_ = ε_22_ = ε_33_ = 0
	ε_12_ > 0; ε_13_ = ε_23_ = 0
8	ε_11_ = ε_22_ = ε_33_ = 0
	ε_13_ > 0; ε_12_ = ε_23_ = 0
9	ε_11_ = ε_22_ = ε_33_ = 0
	ε_23_ > 0; ε_12_ = ε_13_ = 0
10	ε_11_ = ε_22_ > 0; ε_33_ = 0
	ε_12_ = ε_13_ = ε_23_ = 0
11	ε_11_ > 0; ε_22_ < 0; ε_33_ = 0
	ε_12_ = ε_13_ = ε_23_ = 0
12	ε_11_ < 0; ε_22_ > 0; ε_33_ = 0
	ε_12_ = ε_13_ = ε_23_ = 0
13	ε_11_ = ε_22_ < 0; ε_33_ = 0
	ε_12_ = ε_13_ = ε_23_ = 0
14	ε_11_ = ε_33_ > 0; ε_22_ = 0
	ε_12_ = ε_13_ = ε_23_ = 0
15	ε_11_ > 0; ε_33_ < 0; ε_22_ = 0
	ε_12_ = ε_13_ = ε_23_ = 0
16	ε_11_ < 0; ε_33_ > 0; ε_22_ = 0
	ε_12_ = ε_13_ = ε_23_ = 0
17	ε_11_ = ε_33_ < 0; ε_22_ = 0
	ε_12_ = ε_13_ = ε_23_ = 0
18	ε_22_ = ε_33_ > 0; ε_11_ = 0
	ε_12_ = ε_13_ = ε_23_ = 0
19	ε_22_ > 0; ε_33_ < 0; ε_11_ = 0
	ε_12_ = ε_13_ = ε_23_ = 0
20	ε_22_ < 0; ε_33_ > 0; ε_11_ = 0
	ε_12_ = ε_13_ = ε_23_ = 0
21	ε_22_ = ε_33_ < 0; ε_11_ = 0
	ε_12_ = ε_13_ = ε_23_ = 0

Trabecular bone is an anisotropic material; its anisotropy may be quantified with a fabric tensor, which indicates how directionally distributed a material is. The Mean Intercept Length (MIL) fabric tensor is used in this study because it is widely used in trabecular bone studies, and it performs slightly better than other fabric measures (Kabel et al., 1999; Zysset, 2003). The magnitude of an eigenvalue of the MIL fabric tensor denotes the proportion of material which is aligned in the direction expressed in the correspondent eigenvector. The fabric tensors are normalized by a trace equal to 3 (Zysset, 2003).

**Figure 1:**
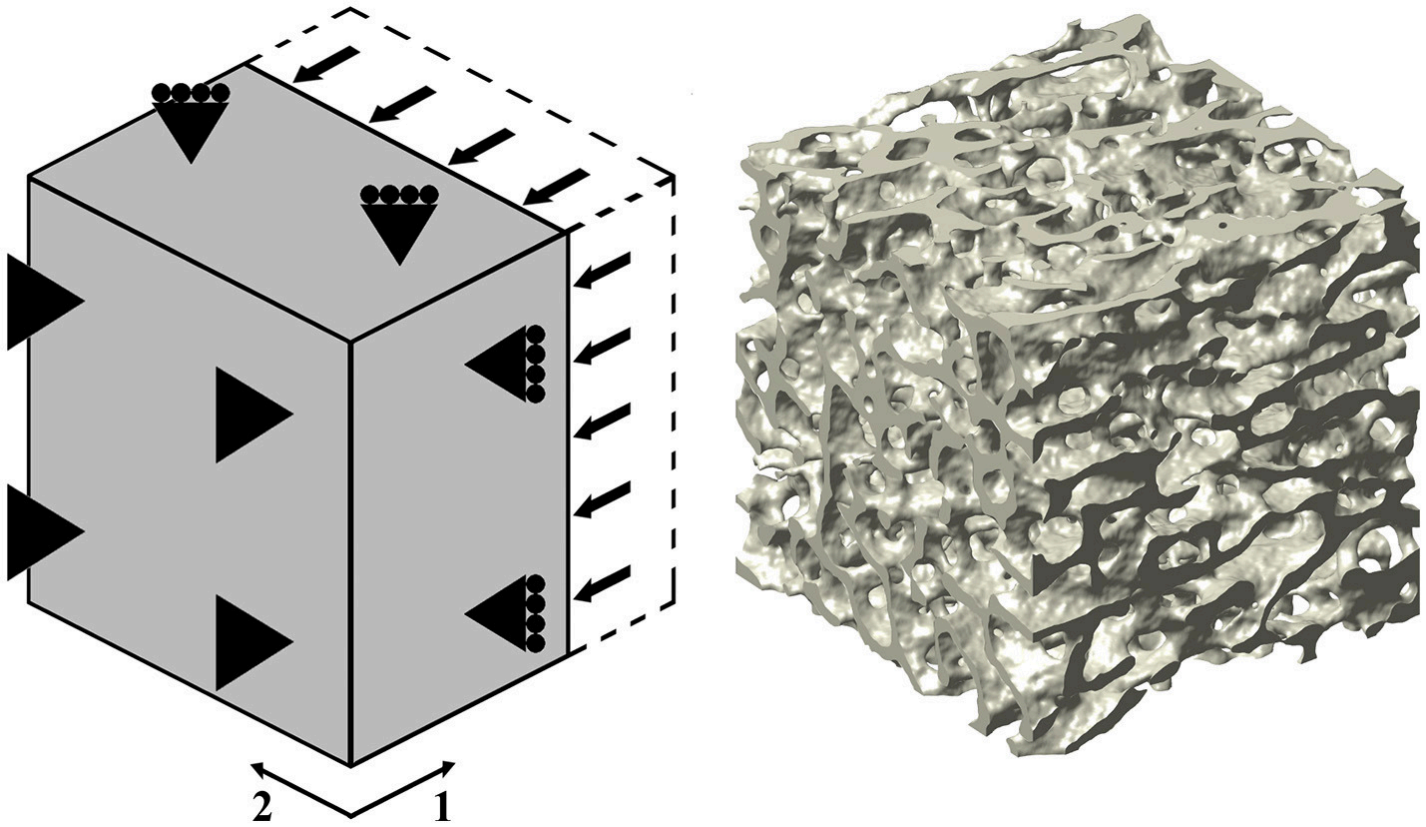
**(Left)** Three-dimensional graphical depiction of the strain-controlled compressive load case in direction 1 (load case 4 in Table 1). **(Right)** Rendered image of one of the used trabecular bone specimens; this particular sample led to a FE mesh of ~21M degrees of freedom.

In this study, 10 out of the 12 samples employed in Levrero-Florencio et al. (2017a) were subjected to 12 additional biaxial strain cases in the normal strain space (Table 1, cases 10–21). Kinematic uniform boundary conditions (i.e., conditions in which displacements, or macroscopic strains, are controlled) were used for all analyses; these are known for providing an upper bound for the macroscopic stiffness tensor and macroscopic yield surface of trabecular bone (Wang et al., 2009; Panyasantisuk et al., 2015). An example of how boundary conditions are implemented can be seen in Figure 1, which corresponds to load case 4 in Table 1. The morphological indices of these samples are shown in Table 2. BV/TV stands for bone volume over total volume and it is a surrogate for density, DOA stands for degree of anisotropy and it is the ratio of the highest to the lowest eigenvalues of the MIL fabric tensor, and SMI stands for structure model index and it ranges from rod- (SMI = 3) to plate-shaped (SMI = 0) microstructure.

**Table 2:** Morphological indices of the 10 used specimens.

**Specimen**	**BV/TV (%)**	**DOA**	**SMI**
1	30.3	2.67	0.52
2	18.1	3.47	1.33
3	14.8	2.65	1.59
4	16.5	2.13	1.37
5	17.7	2.59	1.40
6	22.2	3.47	0.84
7	24.6	2.85	0.88
8	20.3	1.61	1.16
9	23.1	2.10	0.98
10	26.9	2.55	0.79

The 10 samples were aligned with the MIL fabric tensor eigenvectors, with the eigenvalues sorted in descendent order (*m*_1_ > *m*_2_ > *m*_3_). The samples were then meshed with trilinear hexahedra and subjected to the aforementioned strain-controlled load cases; the largest mesh consisted of ~27M degrees of freedom, leading to square sparse stiffness matrices of up to 27M×27M elements. The considered constitutive law at the tissue level was isotropic with coupled plasticity and damage (the former captures irrecoverable deformations while the latter takes accounts for stiffness reduction), meaning that damage and plasticity interact with each other and evolve at the same time; the considered yield surface was Drucker-Prager (Tai et al., 2006; Carnelli et al., 2010; Panyasantisuk et al., 2015) with yield values corresponding to 0.41% strain in tension and 0.83% strain in compression (Bayraktar and Keaveny, 2004). Linear isotropic hardening corresponding to 5% of the undamaged elastic slope (Wolfram et al., 2012; Sanyal et al., 2015) was used. At the tissue level, damage evolution was assumed to be isotropic and it was obtained from Schwiedrzik and Zysset (2013, 2015). The maximum damage was capped at 0.9 (90% isotropic stiffness reduction) to avoid numerical difficulties related to the loss of positive-definiteness of the stiffness matrix; this was performed by using

(4) $$D(\varepsilon^{p})=D_{\text{max}}\left(1-{\text{e}}^{-k_{p}\varepsilon^{p}}\right)$$

where ε^*p*^ = ||**ε**^*p*^|| is the accumulated plastic strain, *D*_max_ is the maximum damage, and *k*_*p*_ is a parameter obtained from Schwiedrzik and Zysset (2015).

The μFE simulations were run on a Cray XC30 supercomputer hosted by ARCHER (UK National Supercomputing Service), with an *in-house* version of ParaFEM (Smith et al., 2013; Levrero-Florencio et al., 2017b) which solves implicit quasi-static finite strain elastoplasticity problems in a highly scalable message passing interface-based (MPI) parallel fashion. Each simulation took from 40 to 120 min when using 1,920 cores, depending on the considered load case, with biaxial compression-compression load cases taking the longest. In order to improve the convergence aspect of the local (constitutive level, i.e., at each integration point) Newton–closest-point projection method (Newton-CPPM), two additional schemes were implemented: (a) a line search as in the primal-CPPM scheme described in Pérez-Foguet and Armero (2002) and (b) an improved trial predictor (Bićanić and Pearce, 1996; de Souza Neto et al., 2008). In the latter scheme, if the first Newton-CPPM fails to converge, it is restarted but this time with the initial guess for stress as **σ**^proj^, which is the stress returned to the frozen yield surface, i.e., no hardening or damage evolution. If these two mechanisms do not work, to ensure that a possible local lack of convergence does not influence the results of the μFE simulations, lack of convergence of the CPPM scheme is broadcasted to all MPI processes in order to cut down the time increment to half of its value. The initial, and maximum, step size corresponded to 0.1% macroscopic strain Frobenius norm and was allowed to decrease to a minimum of 0.001%, if global (structural level, i.e., the global stiffness matrix) or local convergence was not achieved. The global solution scheme employed was Newton-Raphson, and a Jacobi, or diagonally, preconditioned conjugate gradients method was used as the linear algebraic solver.

The macroscopic elastic stiffness tensor was calculated at each time increment by using the homogenization procedure described by van Rietbergen et al. (1995, 1996), in which the macroscopic elastic stiffness tensor 𝔼 is

(5) $$𝔼=\frac{1}{V}\int_{\Omega }\left(1-D_{\mu }\right){𝔼}_{\mu }:𝕄\text{d}V,$$

which, in a FE setting, is equivalent to

(6) $$𝔼=\frac{1}{V}\sum_{i=1}^{{\text{n}}_{\text{els}}}\sum_{j=1}^{{\text{n}}_{\text{ips}}}\left(1-D_{\mu ij}\right){𝔼}_{\mu ij}:{𝕄}_{ij}\text{det}(J_{ij})w_{j},$$

and where *V* is the volume of the cubic region (5 × 5 × 5 = 125 mm^3^), *D*_μ_ is the damage at the solid phase, 𝔼_μ_ is the solid phase undamaged stiffness tensor, n_els_ is the total number of elements in the considered mesh, n_ips_ is the number of integration points in a trilinear hexahedron, det(**J**_*ij*_) is the determinant of the Jacobian of the transformation from normal to natural coordinates, *w*_*j*_ is the weight of the corresponding integration point, and 𝕄 is the local structure tensor, which relates the solid phase strain **ε**_μ_ to the average strain tensor **ε**, such that

(7) $$\varepsilon_{\mu }=𝕄:\varepsilon .$$

This tensor 𝕄 was determined by solving six completely linear FE systems for six macroscopic uniaxial strain cases (three tensile or compressive and three shear). For each of these cases, the tissue strains calculated represent one of the six columns of the matrix projection of 𝕄 (Hollister and Kikuchi, 1992). The assumption made was that the samples are aligned in their orthotropic axes as they were aligned with the MIL fabric tensor eigenvectors (Odgaard et al., 1997). Macroscopic strain points were defined by using the 0.2% strain criterion (Wolfram et al., 2012; Levrero-Florencio et al., 2017a), and it was extended to define further 0.3, 0.4, and 0.5% strain levels. The corresponding damaged slope to calculate these strain points is determined at each time step, depending on the load case. The following is an example for the biaxial tensile case ε_11_ = ε_22_ > 0 (load case 10 in Table 1). Since the macroscopic strains are small, the assumption of linear kinematics can be considered at the macroscale; thus, the homogenized infinitesimal stress can be obtained through the macroscopic infinitesimal strain and the macroscopic stiffness tensor, such as

(8) $$\begin{array}{l} \left\{\begin{array}{c} \sigma_{\text{hom},11}\\ \sigma_{\text{hom},22}\\ \sigma_{\text{hom},33}\\ \sigma_{\text{hom},12}\\ \sigma_{\text{hom},13}\\ \sigma_{\text{hom},23} \end{array}\right\}=\left[\begin{array}{cccccc} E_{1111} & E_{1122} & E_{1133} & E_{1112} & E_{1113} & E_{1123}\\ E_{2211} & E_{2222} & E_{2233} & E_{2212} & E_{2213} & E_{2223}\\ E_{3311} & E_{3322} & E_{3333} & E_{3312} & E_{3313} & E_{3323}\\ E_{1211} & E_{1222} & E_{1233} & E_{1212} & E_{1213} & E_{1223}\\ E_{1311} & E_{1322} & E_{1333} & E_{1312} & E_{1313} & E_{1323}\\ E_{2311} & E_{2322} & E_{2333} & E_{2312} & E_{2313} & E_{2323} \end{array}\right]\left\{\begin{array}{c} \varepsilon_{11}\\ \varepsilon_{22}\\ 0\\ 0\\ 0\\ 0 \end{array}\right\}\\ \;=\left\{\begin{array}{c} E_{1111}\varepsilon_{11}+E_{1122}\varepsilon_{22}\\ E_{2211}\varepsilon_{11}+E_{2222}\varepsilon_{22}\\ 0\\ 0\\ 0\\ 0 \end{array}\right\}, \end{array}$$

where **σ**_hom_ is the homogenized stress tensor, leading to

(9) $$\|\sigma_{\text{hom}}\|=\sqrt{E_{1111}^{2}\varepsilon_{11}^{2}+E_{1122}^{2}\varepsilon_{22}^{2}+E_{2211}^{2}\varepsilon_{11}^{2}+E_{2222}^{2}\varepsilon_{22}^{2}},$$

with the damaged slope being (note that in the considered biaxial cases |ε_*ii*_| = |ε_*jj*_|)

(10) $$K_{\text{dam}}=\sqrt{E_{1111}^{2}+E_{1122}^{2}+E_{2211}^{2}+E_{2222}^{2}}.$$

### 3.2. Theoretical framework of damage

The previously described μFE simulations, together with the homogenization-based multiscale procedure, were used to derive the damaged macroscopic stiffness tensors of the considered samples, for different load scenarios (Table 1) and load levels (0.2, 0.3, 0.4, and 0.5% strain norm). These stiffness tensors were used as data points for a minimization procedure (described in the following subsections), which was used to fit the macroscopic damage behavior to several theoretical damage models: single scalar isotropic formulation, three scalars anisotropic formulation, and isotropic/anisotropic combined formulation with tension/compression asymmetry.

Coupled damage and plasticity were considered for the μFE simulations. However, the focus of this study is on the macroscopic damage behavior of trabecular bone and therefore no plasticity is assumed at the macroscale. This is why, in the following, the total strain **ε** is used instead of the elastic strain **ε**^*e*^.

#### 3.2.1. Basic concepts and description of the baseline model

Let us consider the theoretical framework of elastic degradation by using state variables, from which the different damage constitutive models are derived (Carol et al., 1994, 2002; Murakami, 2012). The starting point of the theoretical framework is the assumption of a Helmholtz free energy potential per unit reference volume ψ of the considered material, from which the state equations are derived. The free energy potential may be expressed as

(11) $$\begin{array}{l} \psi (\varepsilon ,D_{k},R_{k})=\psi^{e}(\varepsilon ,D_{k})+\psi^{D}(R_{k})\\ \;=\frac{1}{2}\varepsilon :𝔼({𝔼}_{0},D_{k}):\varepsilon +\frac{1}{2}\sum_{k=1}^{l}K_{k}R_{k}^{2}, \end{array}$$

where **ε** is the infinitesimal strain tensor, 𝔼 and 𝔼_0_ are, respectively, the damaged and undamaged stiffness tensors, *D*_*k*_ are a set of *l* scalar damage variables; *R*_*k*_ and *K*_*k*_ are, respectively, a set of *l* variables and *l* parameters controlling the size and hardening of the (damage) dissipation potential functions *F*_*k*_ (Equation 16).

Time derivative of Equation (11) yields

(12) $$\dot{\psi }=\frac{\partial \psi }{\partial \varepsilon }:\dot{\varepsilon }+\sum_{k=1}^{l}\frac{\partial \psi }{\partial D_{k}}{\dot{D}}_{k}+\sum_{k=1}^{l}\frac{\partial \psi }{\partial R_{k}}{\dot{R}}_{k},$$

which, when used in the Clausius-Duhem inequality for isothermal processes

(13) $$\sigma :\dot{\varepsilon }-\rho \dot{\psi }\geq 0,$$

gives rise to the dissipation inequality

(14) $$\begin{array}{l} \phi =(\sigma -\rho \frac{\partial \psi }{\partial \varepsilon }):\dot{\varepsilon }-\sum_{k=1}^{l}\rho \frac{\partial \psi }{\partial D_{k}}{\dot{D}}_{k}-\sum_{k=1}^{l}\rho \frac{\partial \psi }{\partial R_{k}}{\dot{R}}_{k}\\ \;=\sum_{k=1}^{l}Y_{k}{\dot{D}}_{k}+\sum_{k=1}^{l}B_{k}{\dot{R}}_{k}\geq 0, \end{array}$$

where ρ is the density of the considered material, $\sigma =\rho \frac{\partial \psi }{\partial \varepsilon }$, $Y_{k}=-\rho \frac{\partial \psi }{\partial D_{k}}$, and $B_{k}=-\rho \frac{\partial \psi }{\partial R_{k}}=K_{k}R_{k}$.

The evolution equations of *D*_*k*_ and *R*_*k*_ are derived from the corresponding dissipation potential functions *F*_*k*_, leading to

(15) $$\dot{D_{k}}={\dot{\gamma }}_{k}\frac{\partial F_{k}}{\partial Y_{k}};\;{\dot{R}}_{k}={\dot{\gamma }}_{k}\frac{\partial F_{k}}{\partial B_{k}},$$

where ${\overset{\cdot }{\gamma }}_{k}$ are indeterminate multipliers. Since *F*_*k*_ also delimit the undamaged region of the considered material, the non-negativeness of Equation (14) is assured (Murakami, 2012). Linear, a priori uncoupled, criteria for *F*_*k*_ are considered in this study (each *D*_*k*_ is related to a single *F*_*k*_), such that

(16) $$F_{k}(Y_{k},B_{k})=Y_{k}-(B_{k}+B_{k,0})=Y_{k}-(K_{k}R_{k}+B_{k,0})\leq 0,$$

where *B*_*k*, 0_ are the initial sizes of *F*_*k*_, i.e., when *R*_*k*_ = 0. These linear functions are considered for the sake of simplicity and also because data on additional strain points is needed so that more complex, non-linear, evolution expressions of the dissipation potentials may be taken into account.

Energy equivalence is adopted here since it automatically induces major symmetry in the stiffness and compliance tensors. This leads to

(17) $$\begin{array}{l} \psi^{e}(\varepsilon ,D_{k})=\frac{1}{2}\varepsilon :𝔼({𝔼}_{0},D_{k}):\varepsilon =\frac{1}{2}\varepsilon :{𝕄}^{\text{T}}(D_{k}):{𝔼}_{0}:𝕄(D_{k}):\varepsilon \\ \;=\frac{1}{2}\varepsilon_{\text{eff}}(\varepsilon ,D_{k}):{𝔼}_{0}:\varepsilon_{\text{eff}}(\varepsilon ,D_{k}), \end{array}$$

where 𝕄 is the fourth-order damage effect tensor which depends on the considered damage formulation, and 𝔸^T^ is defined so that ${𝔸}^{\text{T}}\equiv A_{ijkl}^{\text{T}}=A_{klij}$.

#### 3.2.2. Numerical solution of the damage models

Equations (15, 16) are integrated with Backward Euler. Residual equations for each of the variables to be sought can be formulated, with a format similar to that of CPPM equations of computational plasticity (Armero and Pérez-Foguet, 2002; Pérez-Foguet and Armero, 2002), so that

(18) $$\left\{\begin{array}{c} R_{D,k}\\ R_{R,k}\\ F_{k} \end{array}\right\}=\left\{\begin{array}{c} D_{k,n+1}-D_{k,n}-\Delta \gamma_{k,n+1}\frac{\partial F_{k}}{\partial Y_{k}}{|}_{n+1}\\ R_{k,n+1}-R_{k,n}-\Delta \gamma_{k,n+1}\frac{\partial F_{k}}{\partial B_{k}}{|}_{n+1}\\ Y_{k,n+1}-(K_{k}R_{k,n+1}+B_{k,0}) \end{array}\right\}$$

where *n* stands for the *n*th time increment, and the vertical bar means “evaluated at”.

The resulting set of non-linear equations (Equation 18) can be solved with a numerical scheme, for instance a Newton-Raphson approach. The first step is to calculate the Jacobian of the system, and therefore the residuals (Equation 18) are linearized, leading to (time subscripts are dropped for convenience from now onwards)

(19) $$\left\{\begin{array}{c} 0\\ 0\\ 0 \end{array}\right\}=\left\{\begin{array}{c} \text{d}D_{j}(\delta_{jk}-\Delta \gamma_{k}\frac{\partial }{\partial D_{j}}\frac{\partial F_{k}}{\partial Y_{k}})-\text{d}\Delta \gamma_{k}\frac{\partial F_{k}}{\partial Y_{k}}\\ \text{d}R_{k}-\text{d}\Delta \gamma_{k}\frac{\partial F_{k}}{\partial B_{k}}\\ \text{d}D_{j}\frac{\partial F_{k}}{\partial D_{j}}+\text{d}R_{k}\frac{\partial F_{k}}{\partial R_{k}} \end{array}\right\}.$$

where $\delta_{ij}=\{\begin{array}{l} 0\text{ if }i\neq j\\ 1\text{ if }i=j \end{array}$ is the Kronecker delta. The specific expressions for the derivatives of the Jacobian are presented for each of the considered damage models in the following sections.

The resulting Newton-Raphson scheme to solve for *D*_*k*_, *R*_*k*_, and Δγ_*k*_ is

(20) $${\left\{\begin{array}{c} D_{k}\\ R_{k}\\ \Delta \gamma_{k} \end{array}\right\}}_{m+1}={\left\{\begin{array}{c} D_{k}\\ R_{k}\\ \Delta \gamma_{k} \end{array}\right\}}_{m}-{\left[\begin{array}{ccc} \delta_{jk}-\Delta \gamma_{k}\frac{\partial }{\partial D_{j}}\frac{\partial F_{k}}{\partial Y_{k}} & 0 & -\frac{\partial F_{k}}{\partial Y_{k}}\\ 0 & 1 & -\frac{\partial F_{k}}{\partial B_{k}}\\ \frac{\partial F_{k}}{\partial D_{j}} & \frac{\partial F_{k}}{\partial R_{k}} & 0 \end{array}\right]}_{m}^{-1}{\left\{\begin{array}{c} R_{D,k}\\ R_{R,k}\\ F_{k} \end{array}\right\}}_{m}$$

where *m* stands for the *m*th iteration of the Newton-Raphson scheme.

#### 3.2.3. Damage models

This section describes the three main models, and their variants, used in this study. The first two models, single scalar isotropic model (section 3.2.3.1) and three scalars anisotropic model (section 3.2.3.2) are mainly used to assess the BV/TV and fabric eigenvalue dependencies of macroscopic damage models of trabecular bone. The proposed model (section 3.2.3.3), we believe, is a considerable improvement upon the usually employed single scalar isotropic formulation.

##### 3.2.3.1. Single scalar isotropic formulation

In this simple damage formulation a single scalar damage variable *D* equally affects all the components of the stiffness tensor, i.e., all directions are equally affected by damage. The damage effect tensor is

(21) $$𝕄=(1-D){𝕀}_{\text{sym}},$$

where ${𝕀}_{\text{sym}}=I\underline{\bar{⊗}}I$.

The Helmholtz free energy potential for this model is

(22) $$\psi (\varepsilon ,D,R)=\frac{1}{2}\varepsilon :\;{\left(1-D\right)}^{2}{𝔼}_{0}:\varepsilon +\frac{1}{2}KR^{2},$$

which leads to the following expressions for the conjugate thermodynamic associated variables

(23) $$\begin{array}{l} \sigma ={\left(1-D\right)}^{2}{𝔼}_{0}:\varepsilon \\ Y=-\frac{1}{2}\varepsilon :\frac{\partial 𝔼}{\partial D}:\varepsilon =\varepsilon :(1-D){𝔼}_{0}:\varepsilon \\ B=KR \end{array}$$

and to the following expressions for the derivatives in Equation (20)

(24) $$\begin{array}{l} \frac{\partial }{\partial D}\frac{\partial F}{\partial Y}=0\\ \;\frac{\partial F}{\partial Y}=1\\ \;\frac{\partial F}{\partial D}=\frac{\partial Y}{\partial D}=-\frac{1}{2}\varepsilon :\frac{\partial^{2}𝔼}{\partial D^{2}}:\varepsilon =-\varepsilon :{𝔼}_{0}:\varepsilon \\ \;\frac{\partial F}{\partial R}=-K. \end{array}$$

BV/TV dependence is included in this model by defining $K=K_{0,\text{iso}}\rho^{o}$ and $B=B_{0,\text{iso}}\rho^{p}$, where ρ is the BV/TV of the considered sample, and *o* and *p* are the exponents expressing the BV/TV dependency.

##### 3.2.3.2. Three scalars anisotropic formulation

In the anisotropic damage formulation a damage scalar for each principal direction of the sample is considered (*D*_1_, *D*_2_, and *D*_3_), meaning that each of these three orthogonal directions has a different damage behavior (as previously stated, these orthogonal directions are parallel to the axes of the cubic sample). Since the range of post-elastic strains applied to the sample is relatively small, it is assumed that no rotation of the orthotropic axes occurs. The damage effect tensor is

(25) $$𝕄=({𝕀}_{\text{sym}}-𝔻),$$

where

(26) $$\begin{array}{l} \frac{\partial 𝔻}{\partial D_{1}}=\left[\begin{array}{cccccc} 1 & \alpha & \alpha & 0 & 0 & 0\\ \alpha & 0 & 0 & 0 & 0 & 0\\ \alpha & 0 & 0 & 0 & 0 & 0\\ 0 & 0 & 0 & \beta & 0 & 0\\ 0 & 0 & 0 & 0 & \beta & 0\\ 0 & 0 & 0 & 0 & 0 & 0 \end{array}\right];\;\frac{\partial 𝔻}{\partial D_{2}}=\left[\begin{array}{cccccc} 0 & \alpha & 0 & 0 & 0 & 0\\ \alpha & 1 & \alpha & 0 & 0 & 0\\ 0 & \alpha & 0 & 0 & 0 & 0\\ 0 & 0 & 0 & \beta & 0 & 0\\ 0 & 0 & 0 & 0 & 0 & 0\\ 0 & 0 & 0 & 0 & 0 & \beta \end{array}\right];\\ \frac{\partial 𝔻}{\partial D_{3}}=\left[\begin{array}{cccccc} 0 & 0 & \alpha & 0 & 0 & 0\\ 0 & 0 & \alpha & 0 & 0 & 0\\ \alpha & \alpha & 1 & 0 & 0 & 0\\ 0 & 0 & 0 & 0 & 0 & 0\\ 0 & 0 & 0 & 0 & \beta & 0\\ 0 & 0 & 0 & 0 & 0 & \beta \end{array}\right]. \end{array}$$

in which α and β are parameters which determine how the components of the stiffness tensor are affected by the different damage scalars.

The Helmholtz free energy potential is

(27) $$\psi (\varepsilon ,D_{k},R_{k})=\frac{1}{2}\varepsilon :𝔼({𝔼}_{0},D_{k}):\varepsilon +\frac{1}{2}\sum_{k=1}^{3}K_{k}R_{k}^{2},$$

which leads to the following expressions for the conjugate thermodynamic associated variables

(28) $$\begin{array}{l} \;\sigma =𝔼:\varepsilon =[({𝕀}_{\text{sym}}-𝔻):{𝔼}_{0}:({𝕀}_{\text{sym}}-𝔻)]:\varepsilon \\ \;Y_{k}=-\frac{1}{2}\varepsilon :\frac{\partial 𝔼}{\partial D_{k}}:\varepsilon \\ B_{k}=K_{k}R_{k} \end{array}$$

and to the following expressions for the derivatives in Equation (20)

(29) $$\begin{array}{l} \;\frac{\partial }{\partial D_{j}}\frac{\partial F_{k}}{\partial Y_{k}}=0\\ \;\frac{\partial F_{k}}{\partial Y_{k}}=1\\ \;\frac{\partial F_{k}}{\partial D_{j}}=\frac{\partial Y_{k}}{\partial D_{j}}=-\frac{1}{2}\varepsilon :\frac{\partial }{\partial D_{j}}\frac{\partial 𝔼}{\partial D_{k}}:\varepsilon \\ \;\frac{\partial F_{k}}{\partial R_{k}}=-K_{k}\\ \;\frac{\partial 𝔼}{\partial D_{k}}=-[\frac{\partial 𝔻}{\partial D_{k}}:{𝔼}_{0}:𝕄+𝕄:{𝔼}_{0}:\frac{\partial 𝔻}{\partial D_{k}}]\\ \frac{\partial }{\partial D_{j}}\frac{\partial 𝔼}{\partial D_{k}}=\frac{\partial 𝔻}{\partial D_{k}}:{𝔼}_{0}:\frac{\partial 𝔻}{\partial D_{j}}+\frac{\partial 𝔻}{\partial D_{j}}:{𝔼}_{0}:\frac{\partial 𝔻}{\partial D_{k}} \end{array}$$

Fabric eigenvalue dependencies are included in this model by defining $K_{k}=K_{0,\text{aniso}}m_{k}^{q}$ and $B_{k}=B_{0,\text{aniso}}m_{k}^{r}$, where *m*_*k*_ is the MIL fabric eigenvalue corresponding to the *k*th orthotropic direction of the sample; and *q* and *r* are the exponents expressing the fabric eigenvalue dependency.

##### 3.2.3.3. Combined formulation with tension/compression asymmetry

We propose a combined isotropic/anisotropic damage formulation, which consists of four damage scalars: a single scalar defines the isotropic part of the model (*D*_iso_); and three scalars define the anisotropic part of the model, one for each of the three orthotropic directions (*D*_1_, *D*_2_, and *D*_3_). As in the previous cases, the isotropic damage scalar equally affects all directions, while each of the three orthotropic damage scalars only affect their corresponding orthogonal direction. It is assumed that there is no rotation of the orthotropic axes. The tension/compression asymmetry is included in the damage effect tensor, such that

(30) $$𝕄={𝕀}_{\text{sym}}-{𝔻}_{\text{iso}}-\sum_{i=1}^{3}\left[1+\eta \text{H}(-m_{i}\cdot \varepsilon m_{i}){𝔻}_{\text{aniso},i}\right],$$

where

(31) $${𝔻}_{\text{iso}}=(1-D){𝕀}_{\text{sym}},$$

(32) $${𝔻}_{\text{aniso},i}=\frac{\partial 𝔻}{\partial D_{i}}D_{i}$$

with $\frac{\partial 𝔻}{\partial D_{i}}$ being defined in Equation (26), η is the parameter governing the tension/compression asymmetry, **m**_*i*_ is the *i*^th^ fabric tensor eigenvector, and H(·) is the Heaviside function defined as

(33) $$\text{H}(\cdot )=\{\begin{array}{ll} 1\; & \text{if }(\cdot )>0\\ 0\; & \text{if }(\cdot )\leq 0 \end{array}.$$

The Helmholtz free energy potential for this model is

(34) $$\psi (\varepsilon ,D_{k},R_{k})=\frac{1}{2}\varepsilon :𝔼({𝔼}_{0},D_{k}):\varepsilon +\frac{1}{2}\sum_{k=1}^{4}K_{k}R_{k}^{2},$$

BV/TV and fabric eigenvalue dependencies are included in this model by defining $K_{\text{iso}}=K_{0,\text{iso}}\rho^{o}$; $K_{k,\text{aniso}}=K_{0,\text{aniso}}\rho^{t}m_{k}^{q},k\in \left\{1,2,3\right\}$; $B_{\text{iso}}=B_{0,\text{iso}}\rho^{p}$; and $B_{k,\text{aniso}}=B_{0,\text{aniso}}\rho^{u}m_{k}^{r},k\in \left\{1,2,3\right\}$, where *o* and *p* are the exponents expressing BV/TV dependency of the isotropic part of the model; and *t*, *u*, *q*, and *r* are, respectively the exponents expressing BV/TV and fabric eigenvalue dependencies of the anisotropic part of the model. The rest of expressions in the model are the same to those in section 3.2.3.2.

### 3.3. Fitting of the different damage laws

The different damage constitutive models described in the previous section are fitted to the macroscopic damage response obtained from the homogenization-based multiscale μFE simulations. The constitutive laws were fitted by using a particle swarm optimization scheme (particleswarm, MATLAB R2017b, MathWorks Inc.), followed by a gradient-based scheme (fmincon, MATLAB R2017b, MathWorks Inc.) to enhance the final tuning of the parameters, as it is assumed that when particleswarm finishes, the solution is already within the proximity of a minimum. The minimization problem is thus defined as

(35) $$\text{min }\sum_{i=1}^{n}(\|[{𝔼}_{\text{pred}}(\theta_{s})-{𝔼}_{\mu \text{FE}}]{\|}_{i}{)}^{2},$$

where *n* is the number of samples×load cases×strain levels, which means that the damage results for each sample, each considered load case, and each considered strain level (i.e., 0.2, 0.3, 0.4, and 0.5%) are used in the parameter fitting procedure; ||[𝔼_pred_]|| is the Frobenius norm of the matrix projection of the damaged stiffness tensor predicted by the considered theoretical damage model, ||[𝔼_μFE_]|| is the Frobenius norm of the matrix projection of the damaged stiffness tensor calculated through homogenization, and θ are the *s* different parameters of the considered damage model.

This minimization problem (Equation 35) involves the fitting of parameters which govern the size of the damage dissipation potentials (i.e., the surface containing the elastic regime, in which damage does not develop; it is the damage analog to the yield surface in plasticity), and therefore the solution of the CPPM scheme may involve negative Δγ_*k*_, which are not physical solutions. The CPPM scheme is used in computational plasticity and/or damage contexts to solve the corresponding non-linear equations (Equation 20). If the loading state of a sample is found within the elastic regime (i.e., inside of the yield surface in a plasticity context, or inside the damage dissipation potential in a damage context), no equations need to be solved as plasticity and/or damage related quantities would not further develop. Thus, these undesired values of Δγ_*k*_ will arise only if the loading state of the considered sample is not outside of the damage dissipation potential. In order to avoid these, the minimization problem is modified with a penalty term to avoid such unwanted situations, such that

(36) $$\text{min }\sum_{i=1}^{n}[{\left(\|[{𝔼}_{\text{pred}}(\theta_{s})-{𝔼}_{\mu \text{FE}}]{\|}_{i}\right)}^{2}+\sum_{k=1}^{l}\text{H}(-\Delta \gamma_{i,k})K_{\text{pen}}({\text{e}}^{\left|\Delta \gamma_{i,k}\right|}-1)],$$

where *K*_pen_ is a large (penalty) constant.

The initial choice of a solver not based on gradients is because the addition of this penalty term breaks the *C*^1^ continuity of the functional to be minimized, and its global non-convexity is assumed *a priori*. The specific choice of particle swarm optimization over other methods not based on gradients, such as genetic algorithm, is established on the superior computational efficiency of particle swarm optimization over the genetic algorithm (Panda and Padhy, 2008).

The goodness of the fitting procedure was analyzed with the standard error of the estimate (SEE). This is calculated as

(37) $$\text{SEE}(\%)=100\frac{\sqrt{\sum_{i=1}^{n}{\left(\|[{𝔼}_{\text{pred}}-{𝔼}_{\mu \text{FE}}]{\|}_{i}\right)}^{2}}}{\sqrt{\sum_{i=1}^{n}{\left(\|[{𝔼}_{\text{pred}}-{𝔼}_{0}]{\|}_{i}\right)}^{2}}}.$$

## 4. Results

### 4.1. Evaluation of the μFE results

**Figure 2:**
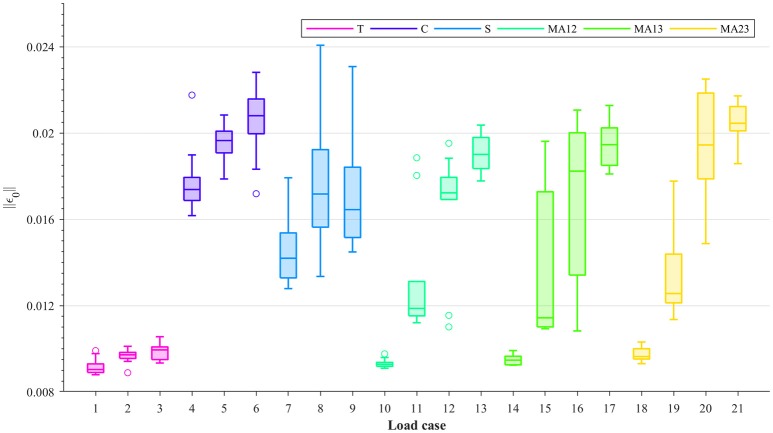
Boxplots showing the Frobenius norms of the macroscopic strain (||**ε**_0_||) at 0.5% strain level, for each load case: uniaxial tension (T), uniaxial compression (C), shear (S), and multi-axial in the normal strain *XY* plane (MAXY).

For all load cases in Table 1, the considered samples were subjected to several strain levels, leading to different damage levels. The resulting macroscopic damaged stiffness tensors and the macroscopic strain Frobenius norms were measured at 0.2, 0.3, 0.4, and 0.5% strain levels by using the 0.2% strain criterion (Wolfram et al., 2012). This theoretically leads to damage and macroscopic strain Frobenius norms being evaluated, respectively, at 0–0.3% (with 0% being considered as macroscopic yield) macroscopic plastic strain Frobenius norms. The macroscopic strain Frobenius norms at 0.5% strain level for each load case are shown in Figure 2 in the form of boxplots. It can be seen from this figure that within each group (T, C, S, or MA), higher macroscopic strain Frobenius norms correspond to compression-dominated load cases (load cases 4–6, 13, 17, and 21 in Figure 2).

**Figure 3:**
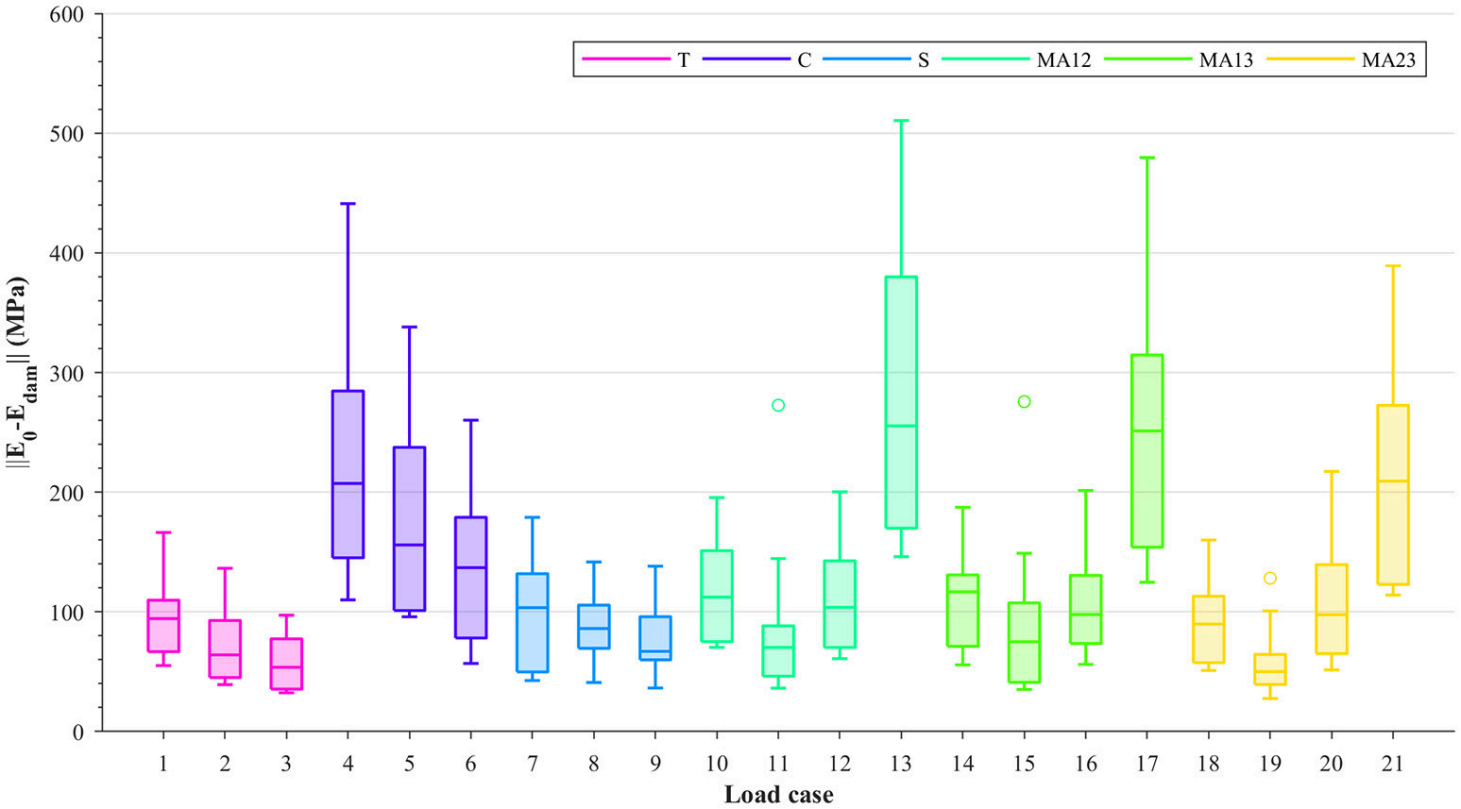
Boxplots showing ||[𝔼_0_ − 𝔼_dam_]|| at 0.5% strain level, for each load case: uniaxial tension (T), uniaxial compression (C), shear (S), and multi-axial in the normal strain *XY* plane (MAXY).

Damage is evaluated by subtracting the damaged stiffness tensor from the undamaged stiffness tensor and calculating the Frobenius norm of its matrix projection (||[𝔼_0_ − 𝔼_dam_]||). The values of these norms for each of the considered load cases are shown in Figure 3; the damage shown corresponds to the 0.5% strain level. Due to the alignment of the samples and ordering of their fabric eigenvalues (*m*_1_ > *m*_2_ > *m*_3_), it can be seen from this figure that within each group (T, C, S, or MA), higher damage values are seen where the fabric tensor eigenvalues are the largest (i.e., load cases 1, 4, 7, and 10–13 in Figure 3). Moreover, higher damage values are also seen in load cases that are compression-dominated (load cases 4–6, 13, 17 and 21). These higher damage values in uniaxial compression, or in compressive-dominated multi-axial load cases, compared to tension load cases indicate a possible tension/compression asymmetry in the damage behavior at the macroscopic level. It is important to mention that, although damage values were measured at the same strain levels according to the 0.2% strain criterion, the macroscopic strain Frobenius norms (Figure 2) were considerably larger in compression than in tension.

Multi-linear regressions in log-log space were performed to establish possible relationships between damage and the micro-architectural indices of the considered samples. These regressions were between ||[𝔼_0_ − 𝔼_dam_]|| at 0.5% strain level, BV/TV, fabric eigenvalues and macroscopic strain Frobenius norms, such as

(38) $$\begin{array}{l} \text{log}(\|[{𝔼}_{0}-{𝔼}_{\text{dam}}]\|)=A+B\text{log}(\text{BV/TV})+C\text{log}(m_{1})\\ \;+D\text{log}(m_{2})+E\text{log}(\|\varepsilon_{0}\|) \end{array}$$

where *m*_1_ and *m*_2_ are the fabric eigenvalues corresponding to directions 1 and 2 (only shear and multi-axial load cases have two directions); *A*, *B*, *C*, *D*, and *E* are the constants in the regression. These regressions were performed separately for the following sets of load cases: uniaxial tension, uniaxial compression, combined uniaxial tension and uniaxial compression, shear, and multi-axial load cases in normal strain space. The results from these regressions can be seen in Table 3. Table 3 shows that both BV/TV and fabric eigenvalues have a significant effect (*p* ≤ 0.05), and that damage expressed as per Equation (38) is directly proportional to the micro-architectural indices, with the slopes for BV/TV being substantially larger than those for the fabric eigenvalues. The coefficients of determination (*R*^2^) show that only the multi-linear model of the multi-axial load cases in normal strain space behaves poorly in comparison to the rest.

**Table 3:** Results from the multi-linear regressions between ||[𝔼_0_ − 𝔼_dam_]|| at 0.5% strain level, BV/TV, fabric eigenvalues, and macroscopic strain Frobenius norms, in log-log space.

**Load case**	***B* (MPa)**	***C* (MPa)**	***D* (MPa)**	***p*-value (BV/TV)**	***p*-value (*m*_*k*_)**	***R*^2^**
T	1.50	0.31		→ 0	0.004	0.91
C	2.03	0.36		→ 0	0.002	0.90
T∪C	1.76	0.66		→ 0	→ 0	0.90
S	1.99	0.53	0.45	→ 0	0.001	0.79
MA	1.71	0.62	0.15	→ 0	0.001	0.63

*Regressions were performed for uniaxial tension (T), uniaxial compression (C), combined uniaxial tension and uniaxial compression (T∪C), shear (S), and multi-axial (MA) in normal strain space*.

The component-wise fraction between the matrix projection of 𝔼_0_ − 𝔼_dam_ at 0.5% strain level and the matrix projection of *E*_0_ (i.e., the *i*-th and *j*-th component of 𝔼_0_ − 𝔼_dam_ is divided by the *i*-th and *j*-th component of 𝔼_0_) leads to the 6 × 6 matrix with components

(39) $${\left[D\right]}_{ij}=\frac{{\left[{𝔼}_{0}-{𝔼}_{\text{dam}}\right]}_{ij}}{{\left[{𝔼}_{0}\right]}_{ij}}.$$

**Figure 4:**
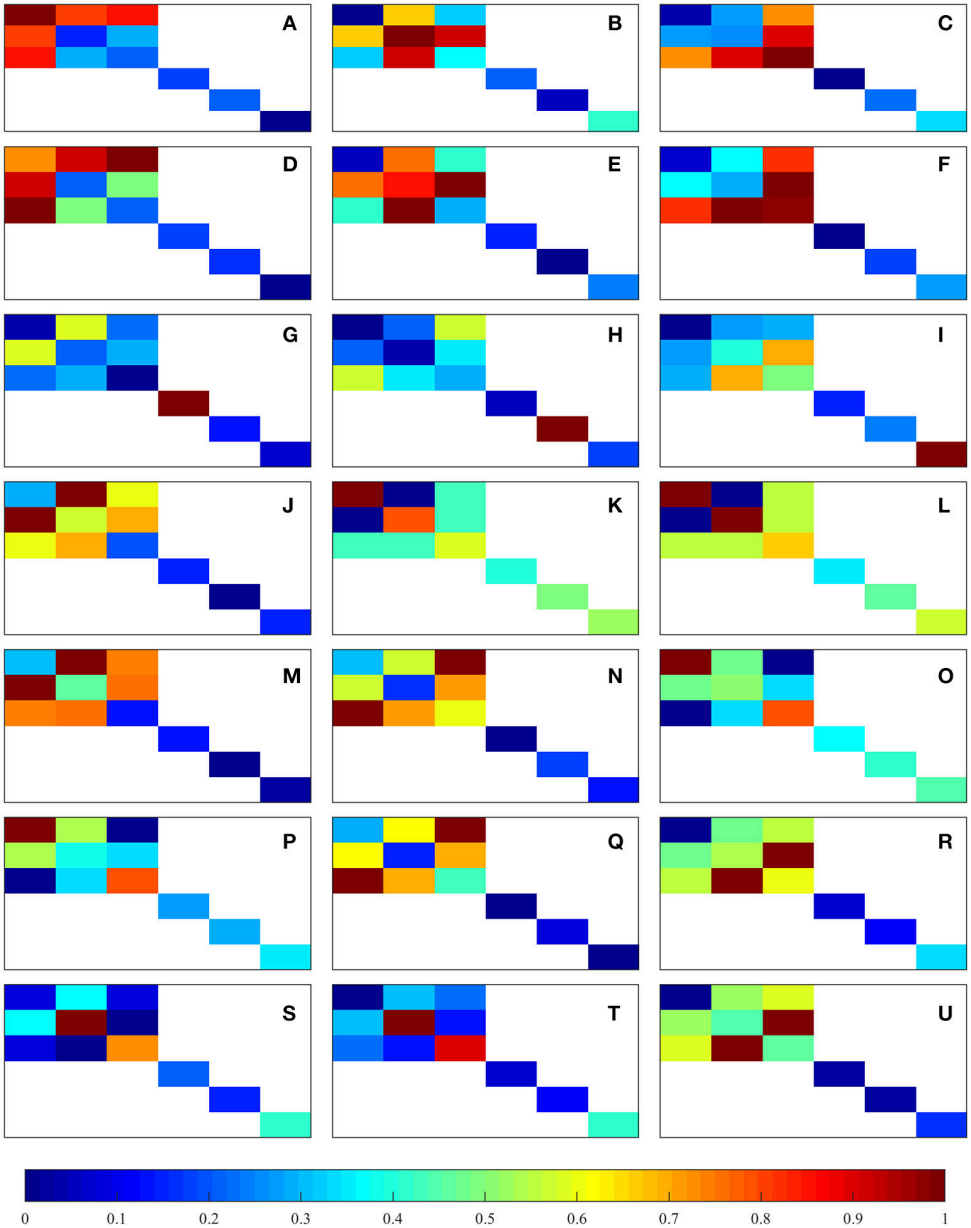
Graphical representation of the normalized component-wise means of [**D**]_*ij*_. The represented load cases are shown in Table 1: **(A–C)** correspond to uniaxial tension, **(D–F)** correspond to uniaxial compression, **(G–I)** correspond to shear, and **(J–U)** correspond to multi-axial in normal strain space.

This matrix depicts the component-wise ratio of the damaged and undamaged coefficients for each sample and load case. The component-wise mean of [**D**]_*ij*_ over all the considered samples was calculated and then normalized from 0 to 1 for each of the considered load cases, forming another 6 × 6 matrix (e.g., the new matrix *i*-th and *j*-th component is the mean of the **D**_*ij*_ components of all the samples); the components in 𝔼_0_ which are zero are ignored and not considered in the normalization, i.e., the non-orthotropic coefficients. The resulting 21 normalized matrices are shown in Figure 4. These plots suggest that macroscopic damage in trabecular bone is actually anisotropic and dependent on the considered load case. In uniaxial tensile and compressive load cases, it can be observed that the normal components of the stiffness tensor which are related to the considered load case are the most affected ones (e.g., in the load case ε_11_ > 0, components *E*_1111_, *E*_1122_, *E*_1133_, and the corresponding symmetric counterparts are more affected than the rest). In shear load cases, the corresponding shear component is the most affected one. Considering multi-axial load cases in normal strain space we find that in tension-tension and compression-compression load cases, the most affected components are in the off-diagonals of the matrix—the components that are related to the plane which is being loaded (e.g., in the load case ε_11_ = ε_22_ > 0, components *E*_1122_ and *E*_2211_ are more affected than the rest); in tension-compression/compression-tension load cases, the most affected components are in the matrix diagonal - the components that are related to the plane which is being loaded (e.g., in the load case ε_11_ = −ε_22_ > 0, components *E*_1111_ and *E*_2222_ are more affected than the rest).

### 4.2. Effect of BV/TV and MIL fabric tensor on the damage behavior

The effect of BV/TV and fabric on the macroscopic damage behavior of trabecular bone was assessed by (1) considering the single scalar isotropic damage model in section 3.2.3.1 with and without considering the effect of BV/TV and then comparing the respective values of SEE; and (2) considering the anisotropic damage model in section 3.2.3.2 with and without considering the effects of BV/TV and fabric eigenvalues and then comparing the respective values of SEE. In the anisotropic scenario, in the case in which fabric eigenvalues were not included, the order of fabric eigenvalues was randomized to maximise the effect of including fabric in the comparison (the ordering no longer corresponds to *m*_1_ > *m*_2_ > *m*_3_; the corresponding stiffness and strain tensors were reordered accordingly). The minimization scheme was run for five times to ensure that a suboptimal solution was not chosen. This comparison is shown in Table 4.

**Table 4:** SEEs, BV/TV, and fabric eigenvalue exponents for the isotropic and anisotropic models.

**Model**	**SEE (%)**	**Exp $K_{0}\cdot \text{BV}/\text{T}{\text{V}}^{k}$**	**Exp $B_{0}\cdot \text{BV}/\text{T}{\text{V}}^{k}$**	**Exp $K_{0}\cdot m_{2}^{k}$**	**Exp $B_{0}\cdot m_{1}^{k}$**
1	37.03				
2	33.03	1.71	1.35		
3	35.21				
4	32.05	1.71	2.35		
5	34.06			0.51	0.37

*Models 1 and 2 are isotropic with and without BV/TV dependency, respectively; models 1, 2, and 3 are anisotropic and: (1) without BV/TV and fabric eigenvalue dependencies, (2) with BV/TV dependency only, and (3) with fabric eigenvalue dependency only*.

Note that the values of SEE of the anisotropic cases are not considerable lower than those of the isotropic cases. This is because even if the damage is higher in the components related to the considered load case, all the components of the stiffness tensor are damaged, and ||[𝔼_0_ − 𝔼_dam_]|| takes into account the reduction of all the components of the stiffness tensor. The exponents that express BV/TV dependency are considerably larger than those expressing fabric eigenvalue dependency.

### 4.3. Macroscopic damage model for trabecular bone

A damage model which incorporates both isotropic/anisotropic damage progression and tension/compression asymmetry was implemented and its efficacy in evaluating the macroscopic damage behavior of trabecular bone was assessed. BV/TV and fabric eigenvalue dependencies were considered; BV/TV dependency was included in the isotropic part of the model while both BV/TV and fabric eigenvalue dependencies were included in the anisotropic part. Tension/compression asymmetry was included as shown in section 3.2.3.3. The SEE and the value of the parameters of the model are shown in Table 5.

**Table 5:** Value of the parameters and SEE of the combined isotropic/anisotropic model with tension/compression asymmetry.

**Parameter**	**Value**
*K*_0,iso_	211.59
*B*_0,iso_	−1.77
*p*	1.98
*l*	1.31
α	0.12
β	0.29
η	−0.25
*B*_0,aniso_	0.00
*K*_0,aniso_	160.62
*u*	4.01
*r*	3.70
*t*	1.75
*q*	0.94
SEE (%)	21.68

This considered model reduces the SEE in more than 15% with respect to the single scalar isotropic model (SEE = 37.03%). Despite the 13 parameters, a considerably larger number in comparison with the two parameters of the isotropic model, the values of some of these parameters suggest that not all of them need to be considered. For instance, the value of *B*_0,aniso_ is very small, which means that these parameters, together with the corresponding exponents expressing BV/TV and fabric eigenvalue dependencies (*u* and *r*) could be ignored, reducing the number of parameters to 10. It is important to point out the negative values of η and *B*_0,iso_.

## 5. Discussion

The macroscopic damage behavior of trabecular bone has been researched in a few studies, but these are usually restricted to uniaxial load scenarios which only permit the assessment of stiffness reduction in the direction of loading (Keaveny et al., 1994b; Zioupos et al., 2008; Garcia et al., 2009). Consequently these studies are unable to provide a comprehensive constitutive model that can be included in whole-bone simulations. This study investigated the possible relationship between damage at the tissue level and the macroscopic multi-axial damage behavior, by employing a homogenization-based multiscale approach to samples with a relatively wide range of BV/TV and fabric tensor eigenvalues, subjected to multiple loading scenarios. The macroscopic damage behavior of trabecular bone was approximated via different continuum damage models: isotropic and anisotropic; with and without BV/TV and fabric eigenvalue dependencies; and with and without tension-compression asymmetry. From the results, it can be concluded that the macroscopic damage behavior of trabecular bone has the following features: BV/TV and fabric eigenvalue dependencies; tension/compression asymmetry; a combined isotropic/anisotropic behaviour. The first two of these features are not unexpected as they play a key role in the evaluation of elastic stiffness (Odgaard et al., 1997; Zysset, 2003), however, the previously unexplored, last feature indicates that damage in trabecular bone is best represented by using both isotropic and anisotropic damage variables. This is likely to be true for most cellular materials.

This study assumed an isotropic model with coupled damage and plastic behavior at the tissue level, which was deemed appropriate as the isotropy assumption at this level is known to result in little to no error in macroscopic results (Cowin, 1997). Isotropic damage at the solid phase level leads to an anisotropic macroscopic damage response with a dependency on the considered load case (Levrero-Florencio et al., 2017a). The variation in the components of the stiffness tensor shows anisotropic damage which depends on the considered load case (Figure 4). Shi et al. (2010) suggested that there is a larger proportion of damaged tissue in the longitudinal trabeculae (direction of loading) for uniaxial load cases, which is in agreement with the results presented here, as the most damaged components of the macroscopic stiffness tensor are always the on-axis components. An issue which may make validation of these results very challenging is the use of kinematic uniform boundary conditions; these boundary conditions are extremely difficult, not to say impossible, to reproduce experimentally, especially for the more complex load cases. Most previous studies involving damage in trabecular bone have used isotropic models (Garcia et al., 2009; Schwiedrzik and Zysset, 2013), which may be acceptable for proportional loading scenarios, but not for changing loads or cyclic loading scenarios, such as those arising during physiological activities.

The results show that the macroscopic strain Frobenius norms were considerably larger in macroscopic compression than in macroscopic tension. This is important in the considered context of damage modeling as the thermodynamic stress-like variables governing damage evolution (*Y*_*k*_) directly depend on the macroscopic strain values, which could explain the higher damage values in compression without the explicit need of modeling tension/compression asymmetry. However, this asymmetry is taken into account because it still leads to a better fit of the damage model and it only consists of one additional parameter. The fact that damage values are higher in compression-dominated load cases compared to tension load cases could be related to the more heterogeneous stress distributions at the solid phase level occurring during macroscopic compression, which includes tensile stresses at the tissue level due to bending and buckling of trabeculae (Stölken and Kinney, 2003). Another important factor to take into account is that the considered model at the tissue level is ductile (i.e., fracture is not incorporated). If fracture was considered at a critical damage threshold, the tension/compression asymmetry would probably be different as tissue damage is more diffused in compression than in tension (Lambers et al., 2014), and therefore a significant decrease of load carrying capacity would occur in tension.

The variation in the components of the stiffness tensor shows anisotropic damage which depends on the considered load case (Figure 4). Shi et al. (2010) suggested that there is a larger proportion of damaged tissue in the longitudinal trabeculae (direction of loading) for uniaxial load cases, which is in agreement with the results presented here, as the most damaged components of the macroscopic stiffness tensor are always the on-axis components. An issue which may make validation of these results very challenging is the use of kinematic uniform boundary conditions; these boundary conditions are extremely difficult, not to say impossible, to reproduce experimentally, especially for the more complex load cases. Most previous studies involving damage in trabecular bone have used isotropic models (Garcia et al., 2009; Schwiedrzik and Zysset, 2013), which may be acceptable for proportional loading scenarios, but not for changing loads or cyclic loading scenarios, such as those arising during physiological activities.

Multi-linear regressions between ||[𝔼_0_ − 𝔼_dam_]||, BV/TV, fabric eigenvalues and macroscopic strain Frobenius norms (from Table 3). It shows that both BV/TV and fabric eigenvalues are statistically significant. The coefficients of determination suggest that only the regression of ||[𝔼_0_ − 𝔼_dam_]|| of the multi-axial load cases in normal strain space behaved poorly in comparison to the others. The slopes of BV/TV are significantly higher than those of fabric eigenvalues, suggesting that BV/TV plays a more important role in these regressions; they also suggest that the higher the BV/TV and fabric eigenvalues, the higher the damage is. Results in Levrero-Florencio et al. (2017a) showed that the damage in the orthotropic coefficients of the macroscopic stiffness tensors do not have significant dependencies on BV/TV or fabric, for each of the considered load cases. In this study the Frobenius norm ||[𝔼_0_ − 𝔼_dam_]|| is used instead, which takes into account the damage of all the components of the macroscopic stiffness tensor. Therefore, the slopes and *p*-values in Table 3 suggest that lower BV/TV samples have a more anisotropic damage behaviour in the sense that the longitudinal trabeculae are more damaged than the oblique, and that higher BV/TV samples have a more isotropic behavior, or are more damaged in general. Even if fabric eigenvalues have a significant effect on ||[𝔼_0_ − 𝔼_dam_]||, the considerably lower slopes suggest that their relevance is significantly lower than that of BV/TV.

The standard errors of the estimate (SEE) and the exponents with respect to BV/TV and fabric eigenvalues of five different damage models indicate that the SEEs are not substantially different in all these considered models, this is because, despite the anisotropic damage behavior, all the components of the stiffness tensor are damaged (Levrero-Florencio et al., 2017a), suggesting that while a combined isotropic and anisotropic model is most suitable for simulating the macroscopic damage behavior of trabecular bone, an isotropic model is not necessarily poor. The SEEs of the models with dependencies are not substantially lower to those without the dependencies, suggesting that the considered BV/TV and fabric eigenvalue dependencies may not be needed. Nonetheless, the results of the multi-linear regressions (Table 3) show significance of BV/TV and fabric eigenvalues when modeling damage. Furthermore, since these five assessed damage formulations only partially model some of the features of the macroscopic damage behavior of trabecular bone mentioned earlier, the dependencies are maintained in the combined isotropic/anisotropic model with tension/compression asymmetry.

It is apparent that the model with a combined isotropic/anisotropic behavior and tension/compression asymmetry is a substantial improvement over the single scalar damage formulation since the SEE is reduced by more than 15% (Table 5). Nonetheless, it is important to mention that this model has 13 parameters instead of 2, though the value of the parameter *B*_0,aniso_ indicates that this parameter and the associated exponents expressing BV/TV and fabric eigenvalue dependencies can be ignored. The negative value of η suggests that if tension-dominated cases had similar strains to those in compression-dominated cases, the damage values would be higher in tension, as a negative value of η implies crack-closure, which is expected as bone could be considered a quasi-brittle material (Hambli, 2013; Mayya et al., 2016). The negative value of *B*_0,iso_ suggest that, when modeling the damage progression with a linear model, there is an initial presence of damage, which has been previously observed in Levrero-Florencio et al. (2017a) (the intercepts of the *y*-axis of the damage-accumulated plastic strain plots are not zero).

This study has a number of limitations. As previously mentioned, bone at the solid phase level is assumed to be ductile, i.e., while reduction in stiffness due to damage is included, fracture is not. This is perhaps appropriate for the considered level of loading, but it is indeed not applicable if large strains are applied, as complete fracture of trabeculae can occur. Nawathe et al. (2013) shows that ductile tissue behavior overestimates the experimental yield properties. Another limitation, previously stated in Levrero-Florencio et al. (2017a), is that although there is plenty of experimental data on uniaxial load cases (Keaveny et al., 1997; Bayraktar and Keaveny, 2004; Sanyal et al., 2012; Manda et al., 2016), these physical experiments do not allow evaluation of stiffness for samples subjected to different load cases and the effect of loading in one direction on the behavior in the others. Therefore, a study completely based on numerical simulations is the only alternative even though the results cannot be currently validated experimentally. The use of kinematic uniform boundary conditions in the μFE analyses could also be considered a limitation, as they are known for providing an upper bound of the stiffness tensor (Pahr and Zysset, 2008; Wang et al., 2009) or macroscopic yield (Panyasantisuk et al., 2015), and may also affect the damage morphology when compared to the *in situ* case (Daszkiewicz et al., 2017). We also assume that the orthotropic directions do not rotate during loading, which may be a valid assumption for the considered range of strains.

Use of a large number of load cases (21) and samples (10) shows that the evolution of the damaged macroscopic stiffness tensor is based on the loading history. By examining relationships between bone microstructural indices (such as BV/TV and fabric) with macroscopic damage constitutive laws, we show that the proposed combined isotropic/anisotropic damage law with tension/compression asymmetry is a viable superior alternative to the widely used single scalar isotropic damage formulation as it reduces the fitting error from 37 to 22%; it does, however, require specification of a larger number of material parameters. The relationships of damage progression with bone's micro-architectural indices (density and fabric) developed in this study provide an approach for the creation of macroscale continuum models that incorporate damage and will, therefore, improve clinical predictions of the behavior of bone and bone-implant systems.

## Author contributions

FL-F designed the study, performed the FE simulations and parameter fittings, and analyzed the data; PP contributed to the design of the study. Both authors contributed to the critical writing and revision of the manuscript.

### Conflict of interest statement

The authors declare that the research was conducted in the absence of any commercial or financial relationships that could be construed as a potential conflict of interest.
